# Clinical Features and Surgical Management of Bile Duct Cyst in Adults

**DOI:** 10.1155/2019/2517260

**Published:** 2019-06-09

**Authors:** Xin Wu, Binglu Li, Chaoji Zheng, Xiaodong He

**Affiliations:** Department of General Surgery, Peking Union Medical College Hospital, Chinese Academy of Medical Sciences and Peking Union Medical College, Beijing, China

## Abstract

**Objectives:**

Bile duct cyst (BDC) is a rare congenital biliary malformation with 20% of cases initially diagnosed during adulthood. Although the incidence of BDC in adults is increasing worldwide, the clinical features of adult BDC remain unclear. The present study was aimed at characterizing the demographic and clinical features of this rare disease.

**Methods:**

We constructed a retrospective database and analyzed records of 106 patients (mean age, 41.0 ± 14.8 years; 18 men (17.0%)) with BDC treated at our institution from May 2012 to October 2018. Data collected included demographic characteristics, clinical manifestations, surgical patterns, and prognoses. We compared the characteristics of patients undergoing their primary BDC resection against those of patients undergoing reoperation. Risk factors for bile duct infection (BDI), a common complication of BDC, were identified using univariate and multivariate analyses.

**Results:**

Abdominal pain was the most common preoperative symptom, but 12 patients (11.3%) were asymptomatic. Ninety-nine patients underwent their primary BDC resection, and 7 patients received reoperation at our hospital. There was no significant difference in the postoperative complication rate between the two groups. Ninety-four patients were followed up for 37.8 ± 23.8 months, and BDI occurred in 33 patients (35.1%). Hilar anastomosis was an independent risk factor for BDI (odds ratio = 3.561; 95%confidence interval = 1.101, 11.517; and *p* = 0.034).

**Conclusion:**

BDC was more frequent in women and abdominal pain was the most common preoperative symptom. The primary reason for reoperation was anastomotic stenosis. Reoperation had similar outcomes to primary resection and may be considered safe and acceptable if performed by a skillful surgeon. BDI was the most frequent postoperative complication with hilar anastomosis being the only independent risk factor. This highlights the importance of proper bile duct flow for surgical outcomes of BDC.

## 1. Introduction

Bile duct cyst (BDC), or biliary dilatation, is a rare congenital malformation that can occur in the intrahepatic biliary tree, extrahepatic bile ducts, or both systems. It is frequently diagnosed in childhood [1]. Approximately 25% of BDC cases are discovered antenatally or within the first year of life, and only 20% of patients are initially diagnosed as adults [2–4]. This disorder occurs three times as frequently in women than men [1, 3, 4]. Compared to Asian countries, Western countries have a much lower rate of BDC; the incidence ranges from 1 per 1,000 in Japan to 1 per 2,000,000 in England [3, 5, 6]. Globally, BDC accounts for about 1% of all benign biliary diseases [7]. It was initially described by Douglas in 1852 [8] and first classified by Alonso-Lej et al. in 1959 [9]. Todani et al. modified the classification in 1977 and updated it in 1997 and 2003 [10–12]. Although the Todani classification system has some drawbacks, such as a simplistic classification of intrahepatic lesions and a lack of direct relationship between different subtypes and surgical procedures, it has been widely introduced and adopted.

Improvements in noninvasive bile duct imaging have led to an increase in BDC diagnoses in adult patients worldwide [3, 13]. The demographic characteristics, clinical manifestations, surgical patterns, and prognoses of BDC in adults have been widely debated, and the medical community lacks a clear consensus. In the present study, we report our findings from a large cohort of adult BDC patients. This study provides a deeper understanding of the features and progression of this rare disease in adults.

## 2. Materials and Methods

### 2.1. Patients

We retrospectively reviewed the medical records of patients with bile duct cysts treated at our facility from May 2012 to October 2018. Patients were selected based on the following inclusion criteria: (I) BDC was diagnosed by preoperative imaging, (II) the patient underwent operation at our hospital and was at least 18 years old, and (III) the medical record was complete. Patients were excluded if they had bile duct dilatation secondary to tumors, calculosis, or stenosis. Clinical data were compiled from both inpatient and outpatient medical records by two independent physicians, and any discordance was resolved by discussion. A retrospective database containing demographic characteristics, laboratory examinations, imaging tests, operation details, and prognoses was constructed and analyzed.

### 2.2. Treatment

The surgical approaches used in primary resection patients were as follows: cholecystectomy, cyst excision, and Roux-en-Y hepaticojejunostomy for Todani types I and IVa; cyst excision and duodenal repair for Todani type III; and cholecystectomy and hepatectomy for Todani type V. In the present study, all type V patients included had main intrahepatic lesions located in the left liver. In order to preserve liver function and ensure proper hepaticojejunostomy, only left hepatectomy was performed. For reoperation patients, surgical patterns were the maximal removal of cysts and stones and reconstruction of anastomosis. Ductal membranes/stenosis was excluded at the time of primary BDC resection. Intrahepatic lithiasis was routinely searched for and cleared. The level of the biliodigestive anastomosis mainly depended on the upper edge of the BDC, because radical excision was preferred. Single-layer choledochojejunostomy was performed with absorbable 4-0 sutures. If the anastomosis was proximal to the origin of the common hepatic duct and within 0.5 cm from the liver, it was defined as hilar anastomosis. In this situation, hilar ductoplasty would be performed prior to the Roux-en-Y hepaticojejunostomy.

Outpatient interviews, e-mails, and telephone calls were used for follow-up. All patients were suggested to be evaluated at our hospital during postoperative follow-up, but some patients from other provinces were followed at their local hospitals. All patients were followed up every 3 months during the first postoperative year, every 6 months during the second, and annually thereafter. Reexamination included blood test, ultrasound, computed tomography (CT), and magnetic resonance cholangiopancreatography (MRCP).

### 2.3. Definitions

Postoperative complications were defined as abnormal events recorded in the 30 days following surgery and classified by the Clavien-Dindo classification of surgical complications [14]. Bile duct infection (BDI) was diagnosed based on the presence of fever, chill, and an abnormally increased leukocyte count and bilirubin level with or without abdominal pain. Abnormal liver function was determined by a rise in alanine aminotransferase beyond 40 U/L, or a rise in gamma-glutamyl transpeptidase beyond 45 U/L.

### 2.4. Statistics

Statistical analysis was carried out by an independent statistician using the Statistical Package for Social Sciences software (version 19.0, IBM Corp., Armonk, NY, USA). Categorical variables were presented as an absolute number or frequency. Continuous variables had been confirmed as being normally distributed before presentation and were shown as the mean ± standard deviation. Differences between study groups were analyzed by Student's *t*-test, *χ*^2^ test, or Fisher's exact test as appropriate. Logistic multivariate regression analysis was performed to identify independent risk factors for BDI. A *p* value < 0.05 was considered statistically significant.

### 2.5. Ethics

This study was approved by the Peking Union Medical College Hospital Institutional Review Board (SK736). All patients or their legal guardian provided written informed consent for the surgical procedures performed. The requirement of informed consent for the publication of data was waived owing to the retrospective nature of the study.

As an observational study, all the data were presented according to the STROBE criteria [15].

## 3. Results

A total of 119 adult patients with BDC were treated at our institution from May 2012 to October 2018. Twelve patients chose close follow-up instead of operation because of severe underlying diseases and associated surgical risk. One patient had coexisting gastric carcinoma and was transferred to the tumor center for neoadjuvant chemotherapy. The remaining 106 BDC patients who underwent surgery were selected for inclusion in this study.

**Figure 1:**
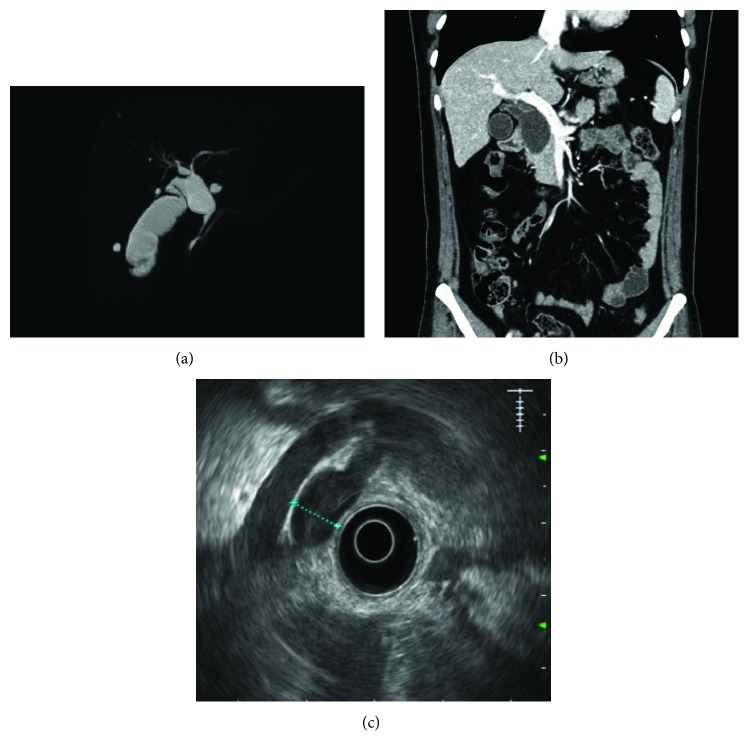
Characteristic findings of imaging modalities in patients with BDC. (a) MRCP reveals anomalous union of the pancreaticobiliary duct, and common bile duct dilatation involving both the left and right intrahepatic bile ducts. (b) CT scanning reveals cystic dilatation of the extrahepatic bile duct. (c) Endoscopic ultrasonography shows common bile duct dilatation. BDC: bile duct cyst; MRCP: magnetic resonance cholangiopancreatography; CT: computed tomography.

The imaging tests carried out for diagnosis were as follows: ultrasonography in 104 patients (98.1%), MRCP in 97 (91.5%), CT in 77 (72.6%), endoscopic ultrasonography in 11 (10.4%), and endoscopic retrograde cholangiopancreatography in 9 (8.5%). Characteristic findings of the diagnostic imaging are shown in Figure 1. As shown in Figure 1(a), the anomalous biliopancreatic junction was quite common in BDC, and this abnormal phenomenon was confirmed in 52 patients (49.1%) in the present study. Patient demographic data, classification, and preoperative symptoms are shown in Table 1. The mean age was 41.0 ± 14.8 years (range = 18 to 80 years), and the ratio of male and female patients was 1 : 4.9. Twelve patients (11.3%) were asymptomatic and were diagnosed incidentally with BDC. Abdominal pain was the most common preoperative symptom.

Eighty-seven patients (82.1%) were diagnosed and underwent their primary resection at our hospital. The remaining 19 patients (17.9%) had previous bile duct operation history. Twelve patients (11.3%) had been treated at the initial operation by derivative surgery and underwent their primary BDC resection at our hospital. Seven patients (6.6%) had undergone initial complete BDC resection at other hospitals and received reoperation at our hospital. Hilar anastomosis was performed in 24 patients. Thirty-four patients (32.1%) had postoperative complications. The reoperation patients had a higher postoperative complication rate than the primary resection patients (71.4%, 5/7, vs. 29.3%, 29/99); however, the difference was not statistically significant (*p* = 0.059). According to the Clavien-Dindo classification, 16 patients were classified as grade I, 11 patients as grade II, 5 patients as grade IIIa, and 2 patients as grade IIIb. A detailed summary of complications and their treatments is shown in Table 2. The demographic characteristics, surgical details, underlying diseases, and complications were compared between primary resection and reoperation patients (Table 3). There was no significant difference between the two groups. A summary of surgical patterns for patients with previous bile duct operation history is shown in Table 4. The most common reason for reoperation was anastomotic stenosis. Postsurgery, 9 patients (8.5%) were pathologically diagnosed with previously undetected coexisting bile duct carcinoma or dysplasia. Their ages were 18, 26, 27, 33, 39, 44, 46, 61, and 65. There was no relationship between incidence and age according to our data.

As of December 2018, 94 patients (88.7%) (87 primary resection and 7 reoperation patients) were followed for 2 to 78 months (mean = 37.8 ± 23.8 months). No postoperative cancer was observed. Definite anastomotic stricture occurred in 1 primary resection patient and 1 reoperation patient, and hepatolithiasis was recorded in 6 primary resection patients. BDI was the most common long-term complication and affected 33 patients (35.1%) (31 primary resection and 2 reoperation patients). There was no significant difference in the long-term complication rate between primary resection and reoperation patients (*p* = 1.000). To analyze risk factors for BDI, patients were divided into two groups for analysis: a BDI-positive group (33 patients) and a BDI-negative group (61 patients). Significant differences were found between the two groups for hilar anastomosis, abnormal liver function one week after surgery, and intrahepatic lithiasis (Table 5). Multivariate logistic regression analysis was performed with select variables, and only hilar anastomosis proved to be independently associated with BDI (OR = 3.561, *p* value = 0.034) (Table 6).

## 4. Discussion

BDC is a congenital bile duct malformation, and the mean age at initial diagnosis in adult patients was between 31.0 and 40.2 years [3, 4, 16]. The present study reported a mean age of 41.0 ± 14.8 years, which is slightly older. Clinical presentations of BDC differ by patient age [17]. The classical triad of jaundice, right upper quadrant pain, and a palpable mass is found more commonly in children than in adults [4]. In contrast, abdominal pain is the most common symptom in adults [2, 18, 19]. Consistently, in the present study, abdominal pain was found in 83.0% of patients, whereas jaundice was only found in 13.2% and abdominal mass in 0.9%. A minority of BDC patients are initially seen as adults, and some can remain asymptomatic for many years [4]. These patients may be diagnosed by an imaging study for an unrelated purpose. In our study, 11.3% of patients were asymptomatic and diagnosed incidentally.

The preoperative diagnosis of BDC is highly reliant on imaging tests. Ultrasonography is the most suitable initial test due to its convenience of operation. However, it is limited in its ability to detect the intrahepatic and distal common bile ducts and in its ability to differentiate a cyst from gall bladder distension in the case of cholecystitis [2, 20]. CT provides more information than ultrasonography about the size, shape, and extent of a cyst. When differentiating the bile duct lesion from malignant disease, an enhanced CT scan is very useful [21]. MRCP is described as the gold standard for diagnosing and staging BDC [1, 3, 20, 22]. It can provide higher clarity images than CT and can reveal cyst anatomy and any existing anomalous biliopancreatic duct junction [22]. At the same time, MRCP is noninvasive and can be performed without the risk of pancreatitis, which is the main complication after endoscopic retrograde cholangiopancreatography. In the present study, after the initial screening by ultrasound, MRCP was the most commonly used deterministic test.

The current recommended surgical pattern for BDC is cholecystectomy and total cyst excision with Roux-en-Y hepaticojejunostomy. However, the ideal surgical procedure cannot be completed in some situations, such as in the case of severe biliary inflammation or incorrect preoperative diagnosis. Some patients not receiving recommended surgical patterns may undergo reoperation. Reoperation may even be required following the standard surgical procedure if complications arise such as anastomotic stenosis and hepatolithiasis. The goals of reoperation are the maximal removal of cysts and stones and reconstruction of anastomosis [23], and reoperation is safe and acceptable if performed by a skilled surgeon.

The incidence of biliary carcinoma in patients with BDC is significantly higher than that in the general population [3, 6]. A Japanese study reported that 16% of patients with BDC or anomalous union of the pancreaticobiliary duct had coincident cancer [24]. Abnormal confluent pancreatic juice is considered a possible cause of preoperative cancer. The incidence of bile duct carcinoma and dysplasia is 8.5% (*n* = 9) in the present study. The low incidence of synchronous cancer may be attributed to the popularization of health examinations in China, which means imaging services are easily available for most people and may promote the early diagnosis and treatment of asymptomatic patients. Some previous studies reported that the incidence of cancer in BDC increased with each decade [25, 26]. However, no relationship between incidence and age was found in the present study. This might be caused by the limited synchronous cancer patient number.

BDI is the most common complication of hepaticojejunostomy. Its incidence is really high in the present study. The high incidence might be associated with a short jejunal loop for the Roux-en-Y hepaticojejunostomy and residual intrahepatic cysts in Todani IVa patients. A suitable surgical approach, healthy diet, and health care pattern are all used to reduce the rate of BDI. In the present study, hilar anastomosis is an independent risk factor for BDI. One possible reason is that retrograde ascending reflux is more likely to enter the intrahepatic bile duct in patients with hilar anastomosis. That causes bacteria and toxins to enter the bloodstream more easily. Radical excision of the bile duct cyst is preferred because incomplete excision is associated with a higher risk of malignancy and worse clinical outcomes [27, 28]. However, this also results in the site of anastomosis being mainly on the upper edge of the cyst. In patients with BDC types Ic and IVa, hilar duct plasty and hilar anastomosis are a routine surgical approach. For these patients, medical education and follow-up are critical for early detection or avoidance of BDI. Interestingly, both reoperation and residual cysts are not associated with BDI in this study. Proper bile duct flow, rather than complete excision, is the most critical factor for surgical outcomes of BDC [6]. Sometimes, complete excision is not achievable in BDC patients with widespread intrahepatic cysts [29]. For these patients and reoperation patients, proper bile duct flow should be achieved to reduce the BDI rate.

There are some limitations to this study. As a result of its retrospective nature, the patient number, variables assessed, and registration information could not be designed in advance. This study is confined to a single institution and may not capture regional trends in BDC. The follow-up time is also relatively short for some patients. Finally, due to a limited patient volume, BDC with different Todani types cannot be compared and discussed separately. Prospective, observational, and multicenter clinical trials will be required to confirm our findings.

## 5. Conclusions

BDC is a rare biliary malformation. This study is consistent with previous reports in that it is more frequent in women and that abdominal pain is the most common preoperative symptom in adult patients. Cholecystectomy, cyst excision, and Roux-en-Y hepaticojejunostomy are the recommended surgical patterns. Our findings show that development of anastomotic stenosis is the most common reason for reoperation. The clinical features and surgical complications of reoperation patients are not significantly different from primary resection patients. BDI is the most common late postoperative complication in all patients, and hilar anastomosis is an independent risk factor for BDI.

## Figures and Tables

**Table 1:** Patient demographic data, classification, and symptoms.

Characteristic	Value
Age (years)	41.0 ± 14.8
Gender	*n* (%)
Male	18 (17.0%)
Female	88 (83.0%)
Todani classification^a^	*n* (%)
I	72 (67.9%)
III	1 (0.9%)
IVa	30 (28.3%)
V	3 (2.8%)
Symptoms and findings	*n* (%)
None	12 (11.3%)
Abdominal pain	88 (83.0%)
Nausea	37 (34.9%)
Fever	28 (26.4%)
Jaundice	14 (13.2%)
Chill	9 (8.5%)
Weakness	1 (0.9%)
Pruritus	1 (0.9%)
Abdominal mass	1 (0.9%)

^a^No patient was classified as Todani type II or IVb.

**Table 2:** Postoperative complications and treatment of patients with BDC.

Complication	*n*	Treatment	*n*	Clavien-Dindo grade
Group 1: primary BDC resection patients (*n* = 99)				
Fever	11	Antipyretic	11	I
Bile duct infection	7	Antibiotic agents	7	II
Ascites	4	CT-guided puncture drainage	4	IIIa
Biliary leakage	2	Bearing drainage tubes^a^	1	I
		Operation	1	IIIb
Hemorrhage	2	Blood transfusion	1	II
		Operation	1	IIIb
Gastroplegia	2	Parenteral nutrition	2	II
Pancreatic leakage	1	Bearing drainage tubes^a^	1	I


Group 2: reoperation patients (*n* = 7)				
Fever	1	Antipyretic	1	I
Bile duct infection	1	Antibiotic agents	1	II
Ascites	1	CT-guided puncture drainage	1	IIIa
Biliary leakage	1	Bearing drainage tubes^a^	1	I
Wound infection	1	Wound dressing	1	I

BDC: bile duct cyst; CT: computed tomography. ^a^The drainage tubes were placed during the index operation.

**Table 3:** Comparison of BDC patients undergoing primary resection or reoperation.

	Primary resection (*n* = 99)	Reoperation (*n* = 7)	*p* value
Male/female (*n*)	17/82	1/6	1.000
Age (years)	41.0 ± 14.8	40.1 ± 15.5	0.878
BMI (kg/m^2^)	22.1 ± 3.2	21.5 ± 4.7	0.650
Hospital stay (d)	16.7 ± 7.3	20.9 ± 11.7	0.170
Operative time (min)	219.8 ± 64.3	209.2 ± 77.9	0.699
Bleeding amount (mL)	207.6 ± 203.7	410.0 ± 406.8	0.330
Hypertension (*n*)	9	0	1.000
Diabetes (*n*)	1	0	1.000
Asthma (*n*)	2	0	1.000
Complications^a^ (*n*)			0.881
I	13	3	
II	10	1	
IIIa	4	1	
IIIb	2	0	

BDC: bile duct cyst; BMI: body mass index. ^a^Complications are classified as the Clavien-Dindo grade.

**Table 4:** Surgical patterns of patients with previous bile duct operation history.

Previous bile duct operation	*n*	Reoperation reason	*n*	Reoperation pattern	*n*
Group 1: initial complete resection patients (*n* = 7)					
CH+CE+HJ	7	AS+hepatolithiasis	3	LH+HJ	3
		Hepatolithiasis	2	Laparotomy+SR	2
		AS	2	LH+HJ	1
				HJ	1

Group 2: initial non resection patients (*n* = 12)					
CH	5	Residual cyst	3	CE+HJ	3
		Hepatolithiasis	2	CE+LLHL+HJ	2
CH+partial CE+CJ	4	AS	4	CE+HJ	4
External biliary drainage	2	Residual cyst	2	CH+CE+HJ	2
Cholecystojejunostomy	1	Residual cyst	1	CH+CE+HJ	1

AS: anastomotic stenosis; CE: cyst excision; CH: cholecystectomy; CJ: cyst-jejunostomy; HJ: hepaticojejunostomy; LH: left hepatectomy; LLHL: left lateral hepatic lobectomy; SR: stone removal.

**Table 5:** Comparison of BDI-positive and BDI-negative patients with BDC.

	BDI-positive (*n* = 33)	BDI-negative (*n* = 61)	*p* value
Male/female (*n*/*n*)	6/27	11/50	0.986
Age (years)	42.3 ± 14.2	41.1 ± 15.4	0.697
BMI (kg/m^2^)	22.3 ± 2.9	22.0 ± 3.5	0.666
Primary resection (*n*)	31	56	1.000
Initial nonresection patients (*n*)	4	8	1.000
Hilar anastomosis (*n*)	13	9	0.007
Residual cyst (*n*)	10	9	0.073
Hepatectomy (*n*)	3	6	1.000
Anastomotic diameter (cm)	1.5 ± 0.9	1.5 ± 0.7	0.999
Abnormal liver function^a^ (*n*)	12	10	0.029
Intrahepatic lithiasis	5	1	0.034
Todani classification (*n*)			0.684
I	20	43	
III	0	1	
IV	12	15	
V	1	2	

BDI: bile duct infection; BDC: bile duct cyst; BMI: body mass index. ^a^One week after surgery.

**Table 6:** Multivariate analysis for risk factors of BDI in patients with BDC.

	*p* value	OR	95% CI
Hilar anastomosis	0.034	3.561	1.101, 11.517
Todani type I+III	0.280	2.546	0.467, 13.890
Intrahepatic lithiasis	0.114	6.749	0.632, 72.110
Residual cyst	0.116	4.255	0.699, 25.909
Abnormal liver function 1 week after surgery	0.155	2.265	0.734, 6.993

BDI: bile duct infection; BDC: bile duct cyst; OR: odds ratio; 95% CI: 95% confidence interval.

## Data Availability

The data used to support the findings of this study are included within the article.
